# Sildenafil does not have a significant effect on the portal vein velocity, cross-sectional area, and congestion index in the dog

**DOI:** 10.3389/fvets.2022.920423

**Published:** 2022-07-19

**Authors:** Christopher R. Tollefson, Marc A. Seitz, Claudio C. Natalini, Alison M. Lee

**Affiliations:** ^1^The Department of Clinical Sciences, Cornell University, College of Veterinary Medicine, Ithaca, NY, United States; ^2^The Department of Clinical Sciences, College of Veterinary Medicine, Mississippi State University, Oktibbeha, MS, United States

**Keywords:** canine, buffer system, liver, sildenafil, portal system, ultrasonography

## Abstract

In veterinary medicine, sildenafil is most frequently used to treat pulmonary hypertension, but has also been investigated and used as a treatment for congenital megaesophagus and ischemic infarcts. With the increasing use, the effects of sildenafil on the portal vasculature in the dog have not been previously evaluated. The purpose of this study was to evaluate the effects sildenafil has on the portal vasculature, which anecdotally may have caused decreased portal vein pressure in an adult dog. The ultrasound cross-sectional area of the aorta, cross-sectional area of the portal vein, and portal vein blood flow velocity were acquired in dogs prior to administration, and 45, 90, and 120 min after oral administration of sildenafil for the treatment of pulmonary hypertension. Thirteen dogs were enrolled in the study. No statistically significant difference was detected between all measured values and the congestion index at all time points. A trend was identified that demonstrated progressively lower portal vein velocity with each evaluation, but this was not significant. Although this study had a small sample size, sildenafil was not shown to have a significant effect on the size or blood flow velocity of the portal vasculature. The hepatic buffer system, designed to maintain a constant blood flow to the liver, may be a contributing factor, but further studies with a larger sample size will be required for further evaluation.

## Introduction

Sildenafil is a selective concentration-dependent, phosphodiesterase-5 (PDE-5) inhibitor that allows for retention of cyclic guanosine monophosphate (cGMP), resulting in vasodilation (1). Additionally, sildenafil amplifies the effects of nitric oxide (NO), another vasodilator, in the target tissues, leading to progressive vasodilation (1). Phosphodiesterase-5 receptors are present on arterial smooth muscle, but in people, PDE-5 messenger ribonucleic acid was found through chromosome mapping in aortic smooth muscle, myocardium, placenta, skeletal muscle, pancreas, and, to a lesser extent, the brain, liver, and lung (2). Sildenafil can weakly inhibit PDE-1 (found in the brain, heart, and bronchial epithelium) (1) and PDE-3 (found in smooth muscles, platelets, cardiac tissues, liver, fat, and corpora cavernosa) (1). Studies have shown sildenafil can be utilized in the treatment of idiopathic megaesophagus and ischemic reperfusion injury (3, 4). In people, sildenafil has been used prior to liver transplant which has had mixed results, showing both improved (5, 6) and aggravated (7) portal vein hypertension. In the dog, identification of portal hypertension is clinically important; however, measurement of the portal vasculature is challenging. Ultrasonographic evaluation of portal vein hypertension is predominantly focused around identifying secondary signs such as peritoneal effusion, portosystemic shunts, decreased portal blood flow velocity, dilation of the portal vein, and dilation of the left gonadal vein (8). Several methods can be utilized to characterize portal vein hypertension including a ratio of the cross-sectional area of the portal vein to the aorta, Doppler blood flow velocity, and congestion index. The congestion index has been shown to be elevated in patients with hepatic disease (9). Because the congestion index incorporates area and blood flow velocity, it is deemed to be a better measurement for diagnosing portal vein hypertension than duplex Doppler evaluation alone (10).

Very limited studies on the effects of sildenafil on the portal system have been performed in veterinary patients. One study performed on rats with medically induced portal hypertension showed sildenafil caused an increase in portal vein pressure and mesenteric blood flow, further convoluting the potential effects sildenafil has on the portal vascular system (11). Despite the studies in human medicine and veterinary patients, the effects of sildenafil on the portal vascular system in dogs remain unclear.

The objective of this study was to evaluate the effect of sildenafil on portal vein blood flow velocity, cross-sectional area, and congestion index in the dog. The authors hypothesized sildenafil will cause an increase in the portal vein to aorta area ratio due to portal venous dilation. Additionally, sildenafil would cause an increase in portal vein blood flow velocity leading to decreased portal vein pressures. With the given increase in portal vein area and increase in velocity, the congestion index of the portal vein would decrease.

## Methods

### Selection and description of subjects

This non-blinded prospective study enrolled client-owned dogs that planned to receive sildenafil as treatment for any presenting condition with a dose of ~2 mg/kg dependent on patient body weight and pill size. The study was performed with institutional IACUC approval and informed owner consent. Patients were enrolled over an ~1-year period. If the dog was already being treated with sildenafil, the dog could not have received a dose in the past 24 h prior to enrollment into the study. Given sildenafil's half-life of 4 h and peak efficacy within 2 h, 24 h was deemed adequate to have negligible remaining effects. If possible, the dog was also fasted for 8 h prior to the ultrasound examinations to reduce gas within the gastrointestinal tract. Exclusion criteria included evidence of liver failure which was characterized by hypoalbuminemia, elevated liver enzymes, hypoglycemia, hypocholesterolemia, elevated bilirubin, and abnormal bile acids or ammonia tolerance. Additional exclusion criteria included known or suspected portosystemic shunts, portal vein obstructions, concurrent administration of beta-blockers, nitrates, propranolol, amlodipine, or vasopressors. These disease processes and drugs were excluded as they cause changes to the portal vein pressures, velocity, and area.

### Data recording and analysis

**Figure 1:**
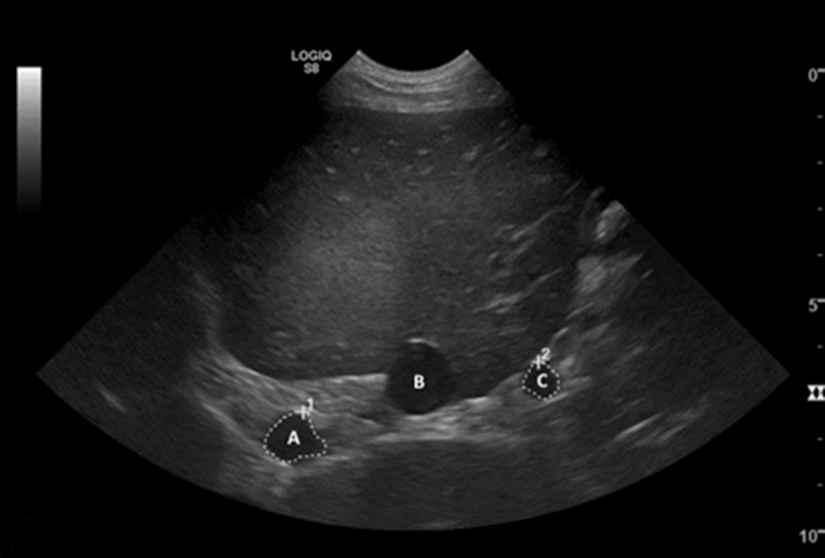
Transverse ultrasound image of the liver. The aorta (A), caudal vena cava (B), and portal vein (C) are visible in cross-section, with the white dashed lines measuring the cross-sectional area of the aorta and portal vein. The area of the portal vein was obtained by acquiring a transverse image of the vessel (perpendicular to its long axis) and traced its outline. From this tracing, the area was calculated by the ultrasound machine software. The same measurement was acquired for the aorta at the same level and the area calculated in the same manner.

Patient inclusion and exclusion was decided by a radiology resident. All images of the study were acquired by a radiology resident or a board-certified veterinary radiologist using a Logiq S8 ultrasound machine (GE Healthcare, Wisconsin, USA) with a C3-10-D Broad Spectrum Micro-Convex (2-11 MHz) transducer (GE Healthcare, Wisconsin, USA). Each dog was placed in left lateral recumbency, and the ultrasound transducer was positioned on the right abdominal wall at the level of the 10th to 12th intercostal space. When necessary, a two-inch square was clipped on the right side of the patient at the 10th to 12th intercostal space on the dorsal one third of the ribs. Alcohol was applied to the skin and coupling gel was applied to the transducer prior to scanning. The aorta and portal vein were scanned while attempting to avoid renal parenchyma or pulmonary parenchyma in the footprint of the transducer (12, 13). Measurement were made with <60 degrees angle of insonation. Attempts were made to acquire the measurements during quiet respiration (Figure 1).

**Figure 2:**
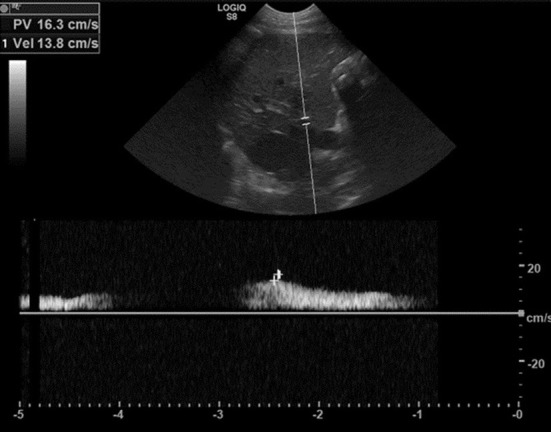
Pulsed-wave Doppler interrogation of the right branch of the portal vein, indicating a portal vein velocity of 13.8 cm/s. The B-mode image with the Doppler gate is shown above. The insonation angle was <60° for evaluation of the right branch of the portal vein in all scans. Angle correction was used as needed. The peak mean velocity was calculated using software inherent to the ultrasound machine. The measurements were obtained prior to administration of sildenafil (sildenafil citrate, ~2 mg/kg as deemed appropriate by the attending clinician, per os, once) (time point 0), then 45, 90, and 120 min after administration.

The blood flow velocity of the portal vein was also acquired for each patient using pulsed wave Doppler. The right branch of the portal vein was identified by moving the transducer one intercostal space cranially for most dogs (9). If the velocity at the right branch was unable to be obtained either due to small patient size or patient compliance, the velocity of the main portal vein was acquired at the same level (Figure 2).

Measurements of the cross-sectional area of the portal vein and the blood flow velocity of the portal vein are required to calculate the congestion index as described by Sartor et al. (5). The cross-sectional area was measured by outlining the perimeter of the portal vein and aorta with the inherent software on the ultrasound machine. These measurements were performed during the examination at each time point previously described. Each time point (prior to administration, 45, 90, and 120 min after administration) corresponds to a data point in the analysis.

### Statistics

All statistics were performed by a board-certified epidemiologist with statistical experience. A paired *t*-test was used to determine if there is a significant change in velocity following administration of sildenafil. An alpha of 0.05, a power of 0.80, and a two-tailed test was applied. Least squares means and 95% confidence intervals were utilized the lower and upper limits are reported for the portal vein velocity.

## Results

Thirteen dogs were enrolled in the study. The average age was 9.5 years (range 2–16 years). Seven spayed females, four neutered males, and two intact males were enrolled. Dog were of various breeds and are listed in table 1. Body weight average was 16.2 kilograms (Kg) and ranged from 2.5 to 29.5 Kg. All patients were being treated for pulmonary hypertension or heartworm disease and had clinical signs ranging from normal to mild elevation of respiratory effort at the initiation of the study.

**Table 1:** Study population.

**Breed**	**Gender**	**Age (years)**	**Weight (Kg)**
Yorkshire Terrier	FS	14	2.5
Labrador Retriever	FS	7	28.5
Shih Tzu	FS	13	5
King Charles Spaniel	MN	7	8.7
Standard Schnauzer	MN	11	12.7
Terrier mix	MN	12	11.9
Shih Tzu	FS	16	10
Mixed	MN	2	29.5
Shih Tzu	FS	14	4
Labrador cross	MI	13	38.2
Jack Russell Terrier	FS	8	7.3
Mixed	FS	3	16.6
Mixed	MI	3	35.5

*Breed, gender, and weight of enrolled patients. FS: spayed female, MN: male neutered male, MI: male intact. Age is reported in years. Weight is reported in kilograms*.

Acquisition of the aorta and portal vein was feasible in all patients. Gas within the duodenum was occasionally present, but did not preclude vascular evaluation. The average results for all time points studied for the portal vein area, aorta area, and portal vein to aorta area ratio are provided in Table 2. A total of 51 data points were acquired during the study period. One 90-min time point data was accidently not saved during the examination, thus excluded from the data calculations. No statistically significant differences were identified in the portal vein area, aorta area, or portal vein to aorta area ratio over time (Table 3). No statistically significant differences were detected at each time point for the portal vein blood flow velocity and congestion index.

**Table 2:** Average portal vein area, aorta, and portal vein area to aorta ratio.

**Time** **(minutes)**.	**Portal vein** **area (cm**^2^**)**	**Aorta area** **(cm**^2^**)**	**Portal vein/** **aorta ratio**
0	0.36 (0.27–0.46)	0.78 (0.52–1.05)	0.49 (0.3–0.69)
45	0.36 (0.27–0.45)	0.81 (0.54–1.07)	0.45 (0.26–0.65)
90	0.32 (0.22–0.41)	0.80 (0.54–1.07)	0.41 (0.22–0.6)
120	0.32 (0.22–0.41)	0.78 (0.51–1.04)	0.47 (0.27–0.66)

*Average portal vein area, aorta area, and portal vein to aorta area ratio for each time examined in 13 dogs. The values provided are the average for the examined time ± the standard error*.

**Table 3:** Average portal vein velocity and congestion index over time.

**Time (minutes)**	**Portal vein velocity** **(cm/s)**	**Congestion index**
0	12.36 (9.95–15.36)	0.029 (0.022–0.037)
45	11.96 (9.63–14.87)	0.030 (0.023–0.038)
90	11.30 (9.07–14.07)	0.028 (0.020–0.035)
120	10.40 (8.37–12.92)	0.030 (0.023–0.038)

*Portal vein velocity and congestion index and 95% confidence intervals. The values provided are the average with the examined time ± the standard error*.

## Discussion

The hypothesis that sildenafil will cause an increase of the portal vein area, portal vein to aorta area ratio, portal vein blood flow velocity, and decrease congestion index in the normal dog was rejected. The results of this study showed there was no statistically significant acute changes of the portal vein parameters following oral administration of sildenafil at any measured timepoint. While on average the portal vein blood flow velocity was progressively lower at each subsequent evaluation, the mean velocity only decreased from 12.36 to 10.40 cm/s (~16%) over the 2-h study window. This change is considered unlikely to be clinically important, as variations in portal velocity of approximately 13% have been reported in normal humans on daily ultrasound examinations (14). Stress of the examination was an unlikely cause for this change in portal vein blood flow velocity as the patients were subjectively calmer with subsequent examinations, but the effect cannot be fully ruled out. The subtle lowering in velocity would be a potential indication for progressive mild portal vein hypertension. This may be explained by a study performed by Colle et al., where sildenafil caused an increase in mesenteric blood flow and portal vein pressure and decreased the mean mesenteric arterial pressure in both the control rats and the experimental rats with a ligated common bile duct (11). In that study the experimental group had a smaller effect than the control group when sildenafil was introduced. Similar conflicting findings were reported in human literature with sildenafil causing an increase and decrease of the portal venous pressures across various studies (5–7). It is possible that sildenafil may cause a mild increase in portal vein pressure in the canine patient by increasing the mesenteric arterial blood flow as with previously reported human and rat studies. This theory cannot be confirmed with this study and may need further investigation with larger animal numbers and more invasive testing. In the aforementioned human studies, patients evaluated had an altered hemodynamic state which likely played a large part of the confounding findings. In the normal patient, the buffer system of the liver becomes activated when there is an alteration in the blood flow to the liver from the portal vein. Minor changes, such as those caused by sildenafil, are likely overcome by this system and thus minimal to no net effects on measured portal vein parameters would be seen. There are numerous causes of portal vein hypertension such as the prehepatic, hepatic, and post hepatic causes discussed earlier. Prehepatic and hepatic disease may cause portal vein hypertension without truly altering the portal vasculature, especially if they are identified and corrected. Further investigation may help identify specific diseases and specific times when sildenafil may be beneficial for each scenario.

The congestion index is one of the more accurate ways to diagnose portal vein hypertension since it incorporates portal vein blood flow velocity and cross-sectional area. In this study, the results were deemed normal and no change was detected with the different time points post-administration.

In this study, the portal vein velocities were on the low end of normal and did not change significantly with the administration of sildenafil. At the authors institution, the described right intercostal approach is frequently employed, but anecdotally, the values are slightly low relative to alternative methods of velocity measurement such as subcostal. This is likely due to the degree of difficulty obtaining an optimal angle of insonation, challenges of measuring the right hepatic branch of the portal vein velocity ultrasonographically, and patient compliance. Additionally, the patients enrolled in the study may have had a component of right sided heart failure which may have yielded a lower portal vein velocity.

Prior studies have used the portal vein diameter for the portal vein to aorta ratios. For this study, the built-in software of the ultrasound machine was utilized to calculate the area. Cross sectional area was chosen as most calculations, such as congestion index, utilize the area rather than just the diameter. Furthermore, cross sectional area may be more accurate since vessels may not always take on a perfect circular cross-sectional shape *in vivo*. Therefore, the values obtained are not directly comparable to the reported normal values, but used as an additional tool to monitor potential portal vein changes over time.

There are three possible explanations for the results of this study. The first possibility is that sildenafil does not have a significant effect on the portal vein in the dog. Although PDE-5 receptors are present on the vascular endothelium, receptors may be in a lower concentration within the portal veins (2). Additionally, as sildenafil causes a decrease in the NO effects, the portal vascular system may not respond as intensely to its effects as the pulmonary vascular system does (1).

The second conclusion to consider is that the effects of the buffer system may be strong enough to suppress the acute changes from sildenafil. Pressure changes within the portal vein cause changes in the hepatic arteries, thus leading to a relatively constant blood supply to the liver (15). Since the hepatic arterial and portal venous vessels converge within the hepatic parenchyma at the sinusoids, the hepatic resistance may then increase, leading to increased portal pressure and decreased velocity. Additionally, sildenafil may cause an increase in blood flow through the mesenteric arteries leading to an increase in blood to the portal system, counteracting any portal vein changes (11).

Finally, all patients enrolled in this study were free of evidence of hepatic disease. Therefore, the effects of sildenafil may have minimal effects on normal patients, but patients with a compromised circulatory system may experience more significant and observable changes.

Ultrasound itself proves to be a difficult modality with which to evaluate portal vein velocity due to the inherent differences between different operators. This was combated by having the same sonographer perform the study at every time point for each dog leading to minimal operator variance. Although this may limit the differences, variables due to the patient such as gas within the intestines, patient anatomy, and patient compliance can also contribute to the difficult nature of this exam.

During the study, the angle of insonation likely varied slightly between scans, which may introduce error in the measurements. For this reason, the insonation angle was kept as low as possible by attempting to measure the portal vein velocity at the right hepatic branch of the portal vein. When visible, the right hepatic branch is angled toward the ultrasound transducer, thus being an ideal area to obtain the portal vein velocity. This was kept as constant and as low as possible, but it still introduced some degree of error in the velocity measurements. Although the right hepatic branch of the portal vein may be an optimal anatomic region to interrogate the velocity of the portal vein, it can be challenging to image with ultrasound, as there is motion during respiration which can yield inaccurate readings.

Variations in dog size may cause error when comparing results between dogs. To correct for this variability, each dog served as its own control with data obtained both before and after the administration of sildenafil. The portal vein velocity can be slightly phasic and the velocity is slightly altered with respiration. This effect was minimalized by making measurement attempts during quiet respiration.

One limitation of this study was the lower than anticipated number of enrolled patients due to a partial shutdown of the hospital during the pandemic, which may potentially have contributed to a Type II error. It is possible that a study with larger animal numbers may have revealed subtle changes that this study was unable to demonstrate. However, the decrease in portal vein velocity over the 2 h study period was subtle (~16%), and further enrollment of dogs was deemed unlikely to yield a change that would be marked enough to have any meaningful clinical relevance.

In conclusion, this study demonstrated sildenafil has a negligible effect on the portal vascular system in the acute phase when administered to dogs.

## Data availability statement

The raw data supporting the conclusions of this article will be made available by the authors, without undue reservation.

## Ethics statement

The animal study was reviewed and approved by Mississippi State University Institutional Animal Care and Use Committee. Written informed consent was obtained from the owners for the participation of their animals in this study.

## Author contributions

CT and AL: conception and design and drafting the article. CT, MS, and AL: acquisition of data. CT, MS, CN, and AL: analysis and interpretation of data and revising article for intellectual content, and final approval of the completed article. All authors contributed to the article and approved the submitted version.

## Funding

This study was funded by the general Resident Research Grant from the American College of Veterinary Radiology in 2019 (grant number: G00004283).

## Conflict of interest

The authors declare that the research was conducted in the absence of any commercial or financial relationships that could be construed as a potential conflict of interest.
